# Agony of choice—selecting chronic lymphocytic leukemia treatment in 2022

**DOI:** 10.1007/s12254-022-00804-x

**Published:** 2022-04-14

**Authors:** Jan-Paul Bohn

**Affiliations:** Department of Internal Medicine V, Hematology and Oncology, Medical University of Innsbruck, Anichstr. 35, 6020 Innsbruck, Austria

**Keywords:** Chemoimmunotherapy, Ibrutinib, Acalabrutinib, Venetoclax, COVID-19

## Abstract

The treatment landscape of chronic lymphocytic leukemia (CLL) has undergone profound change in recent years. Targeted therapies have outnumbered chemotherapy-based treatment approaches demonstrating superior efficacy and tolerability profiles across nearly all CLL patient subgroups in the frontline and relapsed disease treatment setting. Individual selection of these novel agents is rather driven by patients’ comorbidities and personal preferences than fitness and age. Given the high amount of currently licensed novel agents in both treatment-naïve as well as relapsed CLL patients and currently limited evidence from comparative clinical trials, clinicians sometimes appear spoilt for choice when selecting optimal therapy. This short review discusses recent clinical trial data focusing on treatment with targeted drugs and aims to help guide CLL treatment selection in individual patients.

## Introduction

The treatment of patients with chronic lymphocytic leukemia (CLL) has found transformational change in the last decade. Superior in terms of efficacy and tolerability, targeted therapies with inhibitors of Bruton’s tyrosine kinase (BTK), anti-apoptotic protein B‑cell lymphoma 2 (BCL2) and phosphoinositide 3’-kinase (PI3K) strongly shifted in the forefront of commonly accepted treatment algorithms. Notably, CLL patients with high-risk disease such as unmutated immunoglobulin heavy chain variant region (IGHV) status, TP53 aberrations and/or a complex karyotype benefit from treatment with these novel agents compared to chemoimmunotherapy (CIT). However, acquired resistance and drug-associated toxicity continue to pose a challenge in daily clinical practice, typically rendering patients to multiple lines of therapy in the course of this chronic B‑cell malignancy. Optimal selection of these novel agents in individual CLL patients, e.g., in those with high-risk disease features, as well as enrolling the most promising sequence of currently available novel drugs at disease relapse remain topics of ongoing debate, certainly aggravated in times of limited data from randomized comparative clinical trials. This short review discusses recent clinical trial data focusing on treatment with targeted agents and aims to help guide CLL treatment selection in individual patients in the frontline and relapsed/refractory disease setting.

## Selecting frontline treatment

Given the improved progression-free survival (PFS) with targeted drugs as well as prolonged overall survival even in young and fit CLL patients seen with the BTK inhibitor (BTKi) ibrutinib and rituximab compared to CIT with fludarabine, cyclophosphamide and rituximab (FCR) [1], CIT has widely faded into the background in the western hemisphere [2]. Reflecting novel data at ASH 2021 on minimal residual disease (MRD) eradication in the randomized phase III GAIA study [3], appropriate usage of CIT may now even be questioned in the remaining subset of CLL patients with mutated IGHV-status and without TP53 disruption, showing significantly higher rates of undetectable minimal residual disease (uMRD) achievable with the BCL2-inhibitor venetoclax in combination with obinutuzumab (GVe, 86.5% versus 52% with FCR/BR, *p* < 0.0001, *n* = 458). Following the superior data on drug efficacy and tolerability in the era of novel agents, patients’ comorbidities and treatment preferences rather than patients’ age are considered driving criteria for individual therapy selection. As such, continuous BTKi treatment appeals favorably in patients at higher risk of tumor lysis syndrome (TLS) and/or concerns surrounding adequate TLS prophylaxis (e.g., declined renal function, discomfort with requirement of close monitoring/infusion visits) compared to combination treatment with GVe [4]. On the contrary, GVe may be preferred over ibrutinib in patients presenting with cardiac comorbidities, a higher risk of bleeding due to concurrent anticoagulation or basically the desire for a time-limited regimen [5–7]. As recently demonstrated in an ad hoc evaluation of the phase III FLAIR study at ASH 2021 [8], ibrutinib is associated with an 18-times higher risk of sudden unexplained death or cardiac death in young and fit patients with pre-existing arterial hypertension and/or history of cardiac disorders requiring therapy, questioning its usage in this patient subpopulation. Albeit prospective comparative randomized evidence is limited, the currently approved more selective second-generation BTKi acalabrutinib may be at least similarly effective and more favorable in terms of tolerability compared with ibrutinib, particularly as far as cardiac and bleeding side effects are considered [9]. As such, acalabrutinib may be preferred in younger patients with a history of comorbidities predisposing to poor clinical outcome with ibrutinib. High-risk disease with TP53 dysfunction portrays a special situation where GVe may indeed prove inferior to BTKi treatment. At ASH 2021, longer follow-up of the phase III ALLIANCE study demonstrated similar PFS with ibrutinib in patients with or without deletion 17p [10], whereas PFS of patients with deletion 17p was significantly shortened with GVe in the phase III CLL14 study [11]. The randomized phase III CLL16 trial has been launched, investigating acalabrutinib in combination with GVe versus GVe in CLL patients with deletion 17p to better elucidate which treatment regimen to prefer in this patient subgroup (NCT05197192).

## Treatment in relapsed/refractory disease

Given the remarkable efficacy achievable with novel agents, CIT has become obsolete in nearly all patients naïve to novel agents at relapse who typically present with genetically high-risk disease [2]. Most criteria in selecting a targeted drug in the frontline setting, such as patients’ comorbidities and personal preferences, remain valid in the subsequent line treatment setting. Long-term data on both BTKi and venetoclax-based treatments suggest similar efficacy and adequate tolerability in CLL patients relapsed after CIT [5, 12]. Clinical data is limited commenting on whether usage of venetoclax-based regimes is more effective at relapse on BTKi or vice versa, but growing evidence suggests similar clinical outcomes achievable with these agents in either order [13, 14]. PI3K inhibitors (PI3Ki) are generally not used in CLL patients naïve for inhibitors of BTK and BCL2 due to the higher risk of immune-mediated toxicities and infectious complications associated with the currently approved PI3Ki idealisib and duvelisib [15, 16]. Albeit, PI3K inhibitors remain a valuable therapeutic addition in patients refractory or intolerant to BTKi and venetoclax-based regimens [2]. As in front-line therapy, patients with TP53 abnormalities pose a particular challenge when choosing optimal treatment at relapse as novel agents may still not adequately overcome inferior clinical outcomes. Venetoclax indeed has shown promising efficacy in these high-risk patients at relapse when given in monotherapy until disease progression or occurrence of unacceptable toxicity [17]. However, the time-limited approved drug combination with rituximab may pose a higher risk of relapse with rising MRD levels already seen during venetoclax intake in patients MRD positive at the end of treatment in the phase III MURANO study [12]. As such, BTKi may remain treatment of choice in CLL patients with deletion 17p following relapse after CIT. In CLL patients experiencing disease progression on continuous treatment with BTKi, venetoclax-based regimes seem most effective [18]. In the process of transition, however, it is critical to avoid potentially rapid disease progression associated with prompt BTKi discontinuation before venetoclax ramp-up is completed [19]. In CLL patients relapsing after a time-limited approach with venetoclax-based regimes, limited but growing evidence supports retreatment depending on depth and duration of response achieved after first venetoclax exposure [12]. In the scenario of relapsed CLL after a short remission only or even on venetoclax treatment, therapeutic switch to continuous BTKi in naïve patients or alternative BTKis in patients discontinuing previous BTKi treatment due to toxicity is recommended [2]. In patients experiencing disease progression on novel agents, consideration of enrollment in clinical trials is vital with steadily increasing numbers of affected patients.

## Patient management in the COVID-19 pandemic

CLL patients are at high risk of suffering from a severe course of coronavirus disease 2019 (COVID-19) infection, irrespectively of whether they are currently receiving therapy or are closely monitored for disease progression (“watch and wait”) [20]. As such, in CLL patients meeting International Workshop on Chronic Lymphocytic Leukemia (iwCLL) criteria for treatment initiation, it is not recommended to pursue delay of therapy. Safety data on which agent to prefer in the pandemic with regard to the least additional immunosuppressive potential (BTKi versus venetoclax) is conflicting [21, 22]; however, drug combinations with an anti-CD20 antibody may be avoided in case of different therapeutic options [23]. Furthermore, active vaccination is strongly recommended. Although the immune response towards the vaccination may vary in individual patients given the leukemia related immunodeficiency, there is growing evidence that CLL patients may show immune response only after third or even forth booster vaccination [24].

## Conclusion

Targeted drugs have truly revolutionized CLL treatment in the last decade, still continuing to completely supersede usage of CIT. Various novel agents from three major drug classes in terms of inhibitors of BTK, BCL2 and PI3K are currently approved for first- and/or subsequent lines of therapy. Uniformly superior in terms of efficacy and tolerability compared to CIT, particularly in genetically high-risk disease, clinicians and patients appear spoilt for choice which agent to prefer in which clinical setting. As such, nowadays treatment selection is rather driven by patients’ comorbidities and personal preferences than age and fitness status as with former CIT. A history of cardiac disease or a higher risk of bleeding may guide drug selection towards venetoclax-based regimes or more selective second-generation BTKi acalabrutinib associated with a more favorable toxicity profile. Venetoclax-based combinations may be avoided in patients at higher risk for TLS, i.e., due to preexisting renal dysfunction, or those feeling uncomfortable with more frequent hospital visits. Vice versa, patients’ reluctance for an indefinite treatment strategy may attract treatment with venetoclax-based therapy. Albeit, patients with TP53 disruptions remain confronted with generally inferior clinical outcomes, making continuous BTKi intake currently appear the most effective treatment option outside of clinical trials. Prospective comparative randomized clinical trials have been launched helping establish a more tailored use of targeted drugs in individual CLL patients.
